# Evaluation and Assessment of Community Awareness About Coronary Artery Disease in the Aseer Region

**DOI:** 10.7759/cureus.31667

**Published:** 2022-11-19

**Authors:** Zia Ul Sabah, Hasan Ahmed Mohammed Alshorfi, Ali Ahmed Ali AlAsiri, Shahid Aziz, Javed Wani, Humayoun Khan

**Affiliations:** 1 Department of Medicine, King Khalid University, Abha, SAU; 2 College of Medicine, King Khalid University, Abha, SAU; 3 Department of Cardiology, King Khalid University, Abha, SAU

**Keywords:** knowledge, awareness, saudi arabia, aseer, coronary artery disease

## Abstract

Background

Coronary artery disease (CAD), a severe cardiovascular disorder, still remains the major reason for death among adults. In Saudi Arabia, the most common risk factors noticed were hypertension, diabetes, smoking, and dyslipidemia. To date, various therapies have been used for managing CAD, but primary prevention remains the cornerstone to reducing the incidence of CAD-linked mortality and morbidity. The present research aimed to evaluate public awareness levels about CAD in the Aseer region of Saudi Arabia.

Materials and methods

A structured questionnaire was used to assess the demographic variables, information regarding risk factors, and knowledge and awareness about CAD. To analyze the knowledge and awareness of the general population regarding CAD, 26 well-constructed questions were framed and asked. General characteristics like knowledge, awareness, risk factors, signs and symptoms, complications, effects, treatment, and prevention of CAD were recorded by asking questions with different options. The data obtained were then subjected to statistical analysis using SPSS version 20.0 software (IBM Corp., Armonk, NY).

Results

Out of 651 participants, 66.51% were males and 33.48% were females, and 36.40% were aged between 26 and 35 years. Of the participants, 14.13% had a positive family history of CAD, 66.05% had inactive lifestyle habits, and 59.60% did not report any stress. A total of 61.29% were unaware of CAD, but many of them were aware of the risk factors, symptoms, and complications of the disease. A total of 5.529% were suffering from CAD, with a time period of less than one year. Only 1.84% of participants were taking medicines for CAD.

Conclusion

Our study suggested that the community of the Aseer region of Saudi Arabia has meager knowledge and awareness about CAD. Westernized lifestyles and urbanization have caused poor physical well-being in people, leading to increased risk factors for CAD. Thus, we suggest that different educational public health awareness programs should be implemented by the Ministry of Health, Saudi Arabia to decrease the prevalence of these life-threatening diseases.

## Introduction

Noncommunicable diseases (NCDs) accounted for the primary reason of mortality worldwide, with about 71% death rate, among which cardiovascular disease (CVD) has been reported to be the main cause [1]. It has been found that CVD leads to 17.9 million deaths worldwide, out of which coronary artery disease (CAD) was liable for 7.4 million (41.3%) deaths worldwide [2].

In 2018, the World Health Organization (WHO) estimated that countries of the Gulf Cooperation Council (GCC), i.e., Oman, Saudi Arabia, United Arab Emirates, and Kuwait, accounted for 36%, 37%, 40%, and 41%, respectively, contributing the maximum fraction of mortality worldwide because of CVD [2]. Among Middle Eastern countries, the prevalence of CAD was estimated between 5.4% and 13.4% [3]. In Saudi Arabia, the CAD prevalence rate is 5-6% [4]. This high prevalence is because of increased urbanization, rapid socioeconomic growth, a sedentary lifestyle, and increased fast food intake [5].

CAD, a severe cardiovascular disorder, affects about one-third of middle-aged women and 50% of middle-aged men in developed nations [6]. Despite the reduced rate of mortality with CAD, it still remains the major reason for death among adults (>35 years of age) [6].

Various risk factors have been identified leading to CAD such as obesity, diabetes mellitus, smoking, hypertension, stress, dyslipidemia, sedentary lifestyle, fast food intake, old age, and positive family history [7]. In countries of the Middle Eastern region, including Saudi Arabia, the most common risk factors noticed were hypertension, diabetes, smoking, and dyslipidemia [8]. The age group at highest risk was observed to be a decade younger than the mean age (53-63 years) for the disease worldwide [8].

To date, various therapies have been used for managing CAD, but still, prevention remains the cornerstone to reducing the incidence of CAD-linked mortality and morbidity. For preventing the risk factors of CAD, the general public should have adequate knowledge and awareness about the disease. The present research aimed to evaluate public awareness levels about CAD in the Aseer region of Saudi Arabia.

## Materials and methods

The present cross-sectional study was a questionnaire-based study conducted from April 2021 to August 2021. A structured questionnaire was used to assess the demographic variables, information regarding risk factors, and knowledge and awareness about CAD. The validity of the questionnaire was assessed and found to be appropriate (α = 0.84). The study was conducted in accordance with the Declaration of Helsinki and was approved by the Research Ethics Committee of King Khalid University (approval number: ECM 2021-4908). Informed written consent was obtained from all the study subjects prior to their enrolment in this study. The demographic data collected were gender, nationality, area of living, age, education, occupation, marital status, history of smoking, diabetes, hypertension, obesity, and dyslipidemia.

To analyze the knowledge and awareness of the general population regarding CAD, 26 well-constructed questions were framed and asked. The response to all these questions was recorded. General characteristics like knowledge, awareness, risk factors, signs and symptoms, complications, effects, treatment, and prevention of CAD were recorded by asking questions with different options.

The data obtained were then subjected to statistical analysis using IBM SPSS version 20.0 software (IBM Corp., Armonk, NY). Descriptive statistics, i.e., frequencies and percentages, were computed. The comparative analysis was done using chi-square statistical analysis.

## Results

Demographic data were recorded in terms of gender, nationality, region of living, age, education, occupation, marital status, history of smoking, diabetes, hypertension, obesity, dyslipidemia, and place of residence (Table 1).

**Table 1:** Demographic variables and comorbidity status of patients * P-value < 0.05 is significant.

Demographic parameters (n = 651)		Frequency	Percentage	Chi-square	P-value
Gender	Male	433	66.51306	23.923	0.012*
	Female	218	33.48694		
Nationality	Saudi	604	92.78034	18.651	0.001*
	Non-Saudi	47	7.219662		
Region of living	Aseer Region	618	94.93088	11.81	0.034*
	Central Region	15	2.304147		
	North Region	5	0.768049		
	South Region	5	0.768049		
	East Region	3	0.460829		
	West Region	5	0.768049		
Age groups	18-25	164	25.19201	29.971	0.013*
	26-35	237	36.40553		
	36-45	170	26.11367		
	46-55	52	7.987711		
	56-65	28	4.301075		
Education	College/university	346	53.149	25.615	0.05*
	Middle school	46	7.066052		
	High school	228	35.02304		
	Elementary	16	2.457757		
	Illiterate	15	2.304147		
Occupation	Student	177	27.18894	72.817	0.037*
	Civilian job	272	41.78187		
	Businessman	1	0.15361		
	Health practitioner	28	4.301075		
	Housewife	4	0.614439		
	Military	143	21.96621		
	Private sector	3	0.460829		
	Retired	9	1.382488		
	Unemployed	14	2.150538		
Marital status	Married	381	58.52535	17.003	1.819
	Single	198	30.41475		
	Divorced	72	11.05991		
Smoking	Yes	224	34.4086	38.716	0.034*
	No	427	65.5914		
Diabetic	Yes	129	19.81567	29.887	0.04*
	No	453	69.58525		
	Don’t know	69	10.59908		
Hypertension	Yes	115	17.66513	37.525	0.005*
	No	388	59.60061		
	Don’t know	148	22.73425		
Obesity	Yes	112	17.2043	32.81	0.002*
	No	392	60.21505		
	Don’t know	147	22.58065		
Dyslipidemia	Yes	104	15.97542	28.001	0.02*
	No	360	55.29954		
	Don’t know	187	28.72504		
Region of living	Low altitude	327	50.23041	36.817	1.771
	High altitude	324	49.76959		
Where do you live?	Non-urban area	172	26.42089	26.918	0.033*
	Urban area	479	73.57911		

A total of 651 participants were included in the study, with 66.51% males and 33.48% females, having a statistically significant difference (p < 0.05). Of the participants, 92.78% belonged to Saudi Arabia, and 94.93% were from the Aseer region. Most of them belonged to urban areas, with no difference in the altitude of the region. Maximum (36.405%) subjects were aged 26-35 years and the minimum subjects were aged 56-65 years. Maximum subjects (53.14%) were with academic qualification of a diploma, and the minimum subjects were uneducated, with a statistically significant difference. Most of the participants (41.78%) were doing civilian jobs, with the maximum having married marital status. Regarding the habit of smoking, 65.59% were non-smokers, with a statistically significant difference. Maximum participants had a negative history of various systemic diseases, including diabetes, hypertension, obesity, and dyslipidemia, with statistically significant differences (p < 0.05).

Table 2 shows the responses to 26 questions asked to access the knowledge and related factors observed among the general population for CAD. The participants who were having diabetes, hypertension, and dyslipidemia were asked about the time of occurrence and duration of the disease. It was observed that the most common time period they encountered diabetes, hypertension, and dyslipidemia was one to three years, four to six years, and one to three years, respectively, with a statistically insignificant difference (p > 0.05). Of the participants, 14.13% had a positive family history of CAD, and 86.32% of participants had a negative history of chronic kidney disease, with a maximum duration of one to three years. Of the participants, 66.05% had inactive lifestyle habits, and 59.60% did not have any stress. It was found that 61.29% were unaware of CAD. But many of them were aware of gender predilection, risk factors, symptoms, and complications of the disease. In our study, 5.529% were suffering from CAD, with a time period of less than one year. Only 1.84% of participants were taking medicines for CAD.

**Table 2:** Response to questions * P-value < 0.05 is significant.

Questions		Frequency	Percentage	Chi-square	P-value
For how long do you have diabetes?	<1 year	10	1.536098	27.619	0.064
	1-3 years	31	4.761905		
	4-6 years	29	4.454685		
	7-9 years	12	1.843318		
	>10 years	15	2.304147		
For how long do you have hypertension?	<1 year	10	1.536098	29.662	0.071
	1-3 years	19	2.918587		
	4-6 years	30	4.608295		
	7-9 years	8	1.228879		
	>10 years	6	0.921659		
For how long do you have dyslipidemia?	<1 year	20	3.072197	19.001	0.067
	1-3 years	28	4.301075		
	4-6 years	23	3.533026		
	7-9 years	8	1.228879		
	>10 years	2	0.30722		
Family history of coronary artery disease?	Yes	92	14.1321	22.410	0.017*
	Don’t know	1	0.15361		
	No	558	85.71429		
Chronic kidney disease?	Yes	89	13.67127	41.918	0.016*
	No	562	86.32873		
For how long do you have chronic kidney disease?	<1 year	3	0.460829	12.881	0.07
	1-3 years	29	4.454685		
	4-6 years	18	2.764977		
	7-9 years	4	0.614439		
	>10 years	3	0.460829		
Active lifestyle	Yes	221	33.94777	33.960	0.032*
	No	430	66.05223		
Do you have any psychosocial stress?	Yes	263	40.39939	18.051	0.031*
	No	388	59.60061		
Do you have any idea about coronary artery disease?	Yes	252	38.70968	19.240	0.045*
	No	399	61.29032		
How do you define coronary artery disease (CAD)?	It is fluid accumulating in the pericardium preventing the heart from doing complete pumping	36	5.529954	32.994	0.05*
	A mismatch between blood supply and metabolic demand of cardiac muscles mostly due to the narrowing of blood vessels	118	18.12596		
	It is the closure of one or more of the cardiac chambers either atria or ventricles	83	12.74962		
	It is an irregular heart rate due to different causes	32	4.915515		
Which gender is more prone to coronary artery disease?	Male	196	30.10753	24.451	0.037*
	Female	132	20.2765		
	Don’t know	323	49.61598		
Chest pain is a result of	Cardiac causes	348	53.45622	20.623	0.075
	Non-cardiac causes	303	46.54378		
Are recurrent attacks of angina (due to coronary artery disease) related to the severity of the disease?	Yes	442	67.89555	12.881	0.025*
	No	209	32.10445		
Which one of the following do you think is the most common cause of coronary artery disease in our society:	Sedentary lifestyle	229	35.17665	25.191	0.022*
	Nosocomial cause	197	30.26114		
	Aortitis	176	27.03533		
	Connective tissue disorder	49	7.526882		
Which one of the following you would choose as the most appropriate to do when you see a person who starts having symptoms of angina?	Let him take rest and immediately take him to the hospital	324	49.76959	22.771	0.05*
	Take him to the nearest pharmacy and describe the case and take the given medication	127	19.50845		
	The attack is transient no need to take action	79	12.13518		
	Ask if other patient has similar attacks and take their medication and advice	121	18.58679		
Which of the following do you think is a risk factor for coronary artery disease?	Male	184	28.26421	19.991	0.001*
	Positive family history	319	49.00154		
	Dyslipidemia	123	18.89401		
	Diabetes	83	12.74962		
	Hypertension	219	33.64055		
	Age above 45	83	12.74962		
	Smoking	42	6.451613		
	Obesity	35	5.376344		
	Alcohol	30	4.608295		
	Illicit drugs	15	2.304147		
	Sedentary lifestyle	53	8.141321		
	Female gender	153	23.5023		
Do you think when you take a cholesterol-lowering drug it makes you eat as you want a fatty meal?	Yes	152	23.34869	26.910	0.031*
	No	499	76.65131		
Do you think most coronary artery disease patients need surgery?	Yes	234	35.9447	23.001	0.041*
	No	417	64.0553		
Complications of coronary artery disease include	Heart failure	329	50.53763	31.118	0.026*
	Arrhythmia	103	15.82181		
	Stroke	155	23.80952		
	Asthma	30	4.608295		
	Pericarditis	63	9.677419		
	Meningitis	43	6.605223		
	No major complication	68	10.44547		
Do you think that coronary artery disease patients after being managed as per guidelines have a good prognosis?	Yes	136	20.89094	22.817	0.003*
	No	515	79.10906		
Do you have coronary artery disease?	Yes, I have	36	5.529954	31.715	0.042*
	No, I do not have	615	94.47005		
For how long do you have coronary artery disease?	<1 year	11	1.689708	12.615	0.071
	1-3 years	10	1.536098		
	4-6 years	5	0.768049		
	7-9 years	3	0.460829		
	>10 years	1	0.15361		
Are you taking heart disease medication?	Yes	12	1.843318	31.601	0.005*
	No	639	98.15668		

## Discussion

A limited number of studies have been found in the literature on the awareness and knowledge of CAD among the community of Saudi Arabia. The purpose of this study was to evaluate the public awareness levels of CAD in the Aseer region of Saudi Arabia. We observed male predominance in our study, with most of the subjects belonging to the urban population and those who were in college or completed college. Similar results were observed in a previous study [5], in which it was observed that most of the participants were college-going students.

We observed that although 14.13% of participants had a positive family history of CAD (e.g. diabetes mellitus, hypertension, obesity, and dyslipidemia), still participants were not aware of any precautions regarding the control of risk factors of CAD. Still, 66.05% had inactive lifestyle habits and 61.29% were unaware of CAD. They showed meager knowledge about the disease. The percentage was higher in our study as compared to previous studies [9,10].

A comparatively lower level of awareness and knowledge among our study group could be because of scarce awareness programs for the community, less number of community health centers, and less knowledge about health studies.

Related to awareness about risk factors, symptoms, and complications of the disease, study participants were having better knowledge. The results of our study were consistent with a previous study [11]. The studies conducted in other Gulf countries of Dubai [12] and Kuwait [13] also showed similar results. It might be because of the better education level of study participants and most of them belonged to the urban region. Thus, we suggest that awareness can be uplifted via various educational programs, so a better control over CAD, its risk factors, and associated complications can be achieved among the population of Saudi Arabia.

In our study, 14.13% of participants had a positive family history of CAD. Family history was defined as the presence of coronary heart disease (CHD) (i.e., angina, myocardial infarction, and myocardial revascularization) in a first-degree male or female relative (i.e., parents, siblings, and children) before age 55 or 65 years, respectively. Of the participants, 5.529% were suffering from CAD, with a time period of less than one year. Only 1.84% of participants were taking medicines as per the American College of Cardiology (ACC) guidelines for CAD. Studies have demonstrated that a family history of CHD is associated with an approximately 1.5- to 2.0-fold higher risk of CHD independent of conventional risk factors highlighting the contribution of genetic factors to disease susceptibility [14].

We observed that 66.05% had a sedentary lifestyle, which could be an important risk factor for CAD. Similar findings were found in a previous study [15-17], which also advocated that the intake of junk food and a sedentary lifestyle are one of the major modifiable risk factors for CAD.

Due to the rapid development of the economy in Saudi Arabia, lifestyle has changed, posing risk to CAD. Thus, various educational awareness programs should be started by the Ministry of Health, Saudi Arabia in the community to encourage people regarding the role of physical activities to control the incidence of CAD.

## Conclusions

Our study suggested that the community of the Aseer region of Saudi Arabia has meager knowledge and awareness about CAD. Westernized lifestyles and urbanization have caused poor physical well-being in people, leading to increased risk factors for CAD. Thus, we suggest that different educational public health awareness programs should be implemented by the Ministry of Health, Saudi Arabia to decrease the prevalence of these life-threatening diseases. More prospective studies with greater sample sizes should be conducted in the future considering various regions of Saudi Arabia.
